# An Evaluation of the Diagnostic Accuracy of a Panel of Variants in *DPYD* and a Single Variant in ENOSF1 for Predicting Common Capecitabine Related Toxicities

**DOI:** 10.3390/cancers13071497

**Published:** 2021-03-24

**Authors:** Claire Palles, Susan Fotheringham, Laura Chegwidden, Marie Lucas, Rachel Kerr, Guy Mozolowski, Dan Rosmarin, Jenny C. Taylor, Ian Tomlinson, David Kerr

**Affiliations:** 1 Gastrointestinal Cancer Genetics, Institute of Cancer and Genomic Sciences, University of Birmingham, Edgbaston, Birmingham B15 2TT, UK; c.palles@bham.ac.uk (C.P.); LAC757@student.bham.ac.uk (L.C.); 2 Oxford Cancer Biomarkers, The Magdalen Centre, Oxford Science Park, Robert Robinson Avenue, Oxford OX4 4GA, UK; Susan.Fotheringham@oxfordbio.com (S.F.); Guy.Mozolowski@oxfordbio.com (G.M.); 3 Wellcome Trust Centre for Human Genetics, University of Oxford, Roosevelt Drive, Oxford OX3 7BN, UK; marie.lucas1@nhs.net (M.L.); rosmarin.daniel@gmail.com (D.R.); jenny@well.ox.ac.uk (J.C.T.); 4 Department of Oncology, University of Oxford, Old Road Campus Research Building, Roosevelt Drive, Oxford OX3 7DQ, UK; rachel.kerr@oncology.ox.ac.uk; 5 Oxford and National Institute for Health Research Biomedical Research Centre, Unipart House Business Centre, Garsington Road, Oxford OX4 2PG, UK; 6 Cancer Genetics and Evolution Laboratory, Institute of Genetics and Molecular Medicine, University of Edinburgh, Western General Hospital, Crewe Road South, Edinburgh EH4 2XR, UK; Ian.Tomlinson@igmm.ed.ac.uk; 7 Nuffield Division of Clinical and Laboratory Sciences, University of Oxford, Level 6, West Wing John Radcliffe Hospital, Headington, Oxford OX3 9DU, UK

**Keywords:** pharmacogenetics, dihydropyrimidine dehydrogenase, 5-FU

## Abstract

**Simple Summary:**

5-Fluorouracil (5-FU) is a chemotherapy drug that can cause severe toxicity in some patients. A protein, an action molecule in our cells, called dihydropyrimidine dehydrogenase, or DPD for short, is important in clearing 5-FU from the body. Some people have versions of DPD that do not clear 5-FU very well. This causes active drug to stay in the body too long, causing toxicities such as diarrhoea or low levels of blood cells important for fighting infections. Current guidelines identify four sequence changes in the gene that encodes DPD with high level evidence of an impact on protein activity. Our study aims to calculate the frequency of a set of variants identified in patients with DPD deficiency in patients that were part of a clinical trial called QUASAR 2. We go on to test how well the DPD deficiency variants and a set of common variants previously shown to be associated with 5-FU toxicity can predict a person’s risk of 5-Fluorouracil induced toxicity. Our research is important for working out the best way to identify patients at risk of toxicity so high risk patients can be given lower doses of 5-Fluorouracil or be treated with a different drug all together.

**Abstract:**

Efficacy of 5-Fluorouracil (5-FU)-based chemotherapy is limited by significant toxicity. Tests based upon variants in the Clinical Pharmacogenetics Implementation Consortium (CPIC) guidelines with high level evidence of a link to dihydropyrimidine dehydrogenase (DPD) phenotype and 5-FU toxicity are available to identify patients at high risk of severe adverse events (AEs). We previously reported associations between rs1213215, rs2612091, and NM_000110.3:c.1906-14763G>A (rs12022243) and capecitabine induced toxicity in clinical trial QUASAR 2. We also identified patients with DPD deficiency alleles NM_000110.3: c.1905+1G>A, NM_000110.3: c.2846C>T, NM_000110.3:c.1679T>G and NM_000110.3:c.1651G>A. We have now assessed the frequency of thirteen additional *DPYD* deficiency variants in 888 patients from the QUASAR 2 clinical trial. We also compared the area under the curve (AUC)—a measure of diagnostic accuracy—of the high-level evidence variants from the CPIC guidelines plus and minus additional *DPYD* deficiency variants and or common variants associated with 5-FU toxicity. Including additional *DPYD* deficiency variants retained good diagnostic accuracy for serious adverse events (AEs) and improved sensitivity for predicting grade 4 haematological toxicities (sensitivity 0.75, specificity 0.94) but the improvement in AUC for this toxicity was not significant. Larger datasets will be required to determine the benefit of including additional *DPYD* deficiency variants not observed here. Genotyping two common alleles statistically significantly improves AUC for prediction of risk of HFS and may be clinically useful (AUC difference 0.177, sensitivity 0.84, specificity 0.31).

## 1. Introduction

5-Fluorouracil (5-FU)-based adjuvant chemotherapy is commonly used to treat a number of cancers, including colorectal cancer (CRC) following evidence that it effectively increases survival of stage II/III colorectal cancer patients [1,2]. However, its therapeutic ratio is limited by toxicities that arise during treatment in some patients, particularly the elderly. These include myelosuppression, mucositis, diarrhoea and hand foot syndrome (HFS).

Dihydropyrimidine dehydrogenase (DPD), encoded for by the gene *DPYD,* is the rate-limiting enzyme involved in breakdown of 5-FU. Patients with one or more copies of alleles that lower the activity of DPD are at high risk of severe toxicity when treated with 5-FU-based chemotherapy. Approximately 7% of European populations carry a single copy of a *DPYD* decreased function/no function allele [3]. There are also very rare patients with DPD deficiency in whom both parental copies of *DPYD* are mutated [4,5,6,7]. Despite efforts by Offer [8], not all alleles identified in patients with DPD deficiency have had functional assessment in vitro.

Common variants (minor allele frequency (MAF) > 1%) in or near to *DPYD* have also been shown to be associated with risk of toxicity. We previously identified associations between variants NC_000001.10:g97523004G>A (rs12132152) and NM_000110.3:c.1906-14763G>A (rs12022243) and capecitabine induced toxicities using data from QUASAR 2 (QUick And Simple And Reliable 2, randomised study of adjuvant chemotherapy in colon and rectal cancer) [9]. QUASAR 2 also contributed to a meta-analysis that provided statistical evidence for an association between *DPYD* c.1236G>A/HapB3 and 5-FU toxicity [10]. The variant with the strongest effect is rs12132152, estimated to increase risk of global 5-FU toxicity by 3.8-fold [9]. We also identified an association between a variant mapping to *ENOSF1* NC_000018.9:g.683607C>G (rs2612091) and HFS which has been replicated in other samples [11,12]. Enolase Superfamily member 1 (ENOSF1) has several isoforms, whilst one converts L-fuconate to 2-keto-3-deoxy-L-fuconate other isoforms seems to regulate thymidylate synthase (TS) at the transcript and protein level [13]. rs2612091 is in strong linkage disequilibrium (LD) with TYMS variants extensively studied in relation to 5-FU toxicity [14,15]. 

MicroRNAs miR-27a and miR-27b have been shown to repress DPD expression [16]. A common SNP NC_000019.9:g.13947292T>G (rs895819-*MIR27a*) was shown to be associated with early onset severe toxicity in a meta-analysis of 1592 patients [17]. Other candidate genes have also been investigated for associations with 5-FU toxicity but their clinical utility is currently unclear [11,18].

The Clinical Pharmacogenetics Implementation Consortium (CPIC) and the Dutch Pharmacogenetics Working Group (DPWG) have reviewed the literature to identify *DPYD* variants that had been reported in relation to 5-FU toxicity [3,19]. CPIC classified variants as having high, moderate or weak evidence of an effect on DPD phenotype and toxicity [3,19]. Four variants were classified as having high evidence: “no function” *DPYD* alleles NM_000110.3:c.1905 +1G>A (rs3918290) and NM_000110.3:c.1679T>G (rs55886062) and “decreased function” alleles NM_000110.3:c.2846T>A (rs67376798) and NM_000110.3:c.1129–5923C>G”. Statistical evidence suggests that c.2846T>A (rs67376798), c.1905 +1G>A (rs3918290), and 1679T>G (rs55886062) increase the risk of toxicity by at least 4.4-fold and that NM_000110.3:c.1129 5923C>G rs75017182 (HapB3) has a more modest effect on increasing risk of toxicity (hazard ratio (HR) = 1.59 [10,20]. DPWG identify the same four variants as having sufficient evidence to be implemented into clinical care. Innocenti et al. [21] prepared a simple guide for oncologists presenting the evidence for testing for *DPYD* variants before treating with fluoropyrimidines. In many countries there is still a lack of recommended systematic testing. Routine testing for the four variants with high level evidence highlighted by CPIC and DPWG is now recommended in the UK and the PRECISE study [22] demonstrated that a test including 3 of these variants as part of a larger panel (no marker for HapB3 included) provided clinically relevant information to clinicians prior to their prescribing chemotherapy.

Here we aimed to investigate the impact of including additional selected variants on prediction of the spectrum of toxicities associated with treatment with 5-FU. We searched the literature for genetic variants reported in DPD deficient patients (with supporting evidence of an impact on protein activity and toxicity) and common variants of moderate effect (odds ratio > 1.5). Some of these variants had already been genotyped in the QUASAR 2 clinical trial but 12 variants identified in DPD deficient patients were newly genotyped. Comparative receiver operator curve characteristic analysis was performed to compare the impact of adding additional variants to the four variants highlighted by CPIC/DPWG.

## 2. Materials and Methods

We considered genetic markers fulfilling either of these criteria for inclusion in an expanded predictive panel:

1. Statistical evidence of an association with global fluoropyrimidine associated toxicity in ≥1 study with ≥500 patients with an OR/HR of ≥1.5.
2. *DPYD* variant identified in ≥1 patient(s) with DPD deficiency AND ≥ 1 of the following supporting pieces of evidence consistently suggesting a negative impact on the protein activity or where an association with toxicity had been explored evidence of an increased risk of toxicity:
  (a) Analysis of pig DPD crystal structure predicts impact on protein folding or interactions
  (b) Variant allele associated with lower enzyme activity in patient samples or in vitro models (see Table 1).
  (c) No contradictory evidence in studies of 5-FU toxicity.

The QUASAR 2 randomised controlled trial of capecitabine ±bevacizumab has been described previously, as have the genotyping and imputation data available [9,23]. Briefly, participants were stage II/stage III colorectal cancer patients with performance status 0/1. The trial registration number for QUASAR 2 is: ISRCTN45133151 https://doi.org/10.1186/ISRCTN45133151 (access on 15 March 2021). All participants included in this study had consented for use of biological samples and medical records in research. Six variants (NM_000110.3: c.2846C>T (rs67376798), NM_000110.3: c.1905+1G>A (rs3918290), NC_000018.9:g.683607C>G (rs2612091), NM_000110.3:c.1679T>G (rs55886062), NM_000110.3:c.557A>G (rs115232898), NM_000110.3:c.1651G>A (rs777425216)) considered for inclusion had previously been directly genotyped using Illumina arrays (Human Hap 370, Hap 610,Omni 2.5M or Infinium HumanExome (Illumina, San Diego, CA, USA)) or via c(K)ompetitive Allele-Specific PCR (KASP^TM^) (LGC Biosearch Technologies^TM^, Hoddesdon, UK) assays (NM_000110.3:c.1651G>A; rs777425216). 4 additional variants were partially or fully imputed with high confidence (INFO scores >0.8) from the whole genome SNP genotype array data using SHAPEIT (v2.r900) [24], IMPUTE2 (v2.3.2) [25,26] and the UK10K (project to characterise rare and low-frequency variation in the UK population) and 1000 genomes Phase 3 merged reference panel (EGAD00001000776) (NC_000001.10:g.97523004G>A (rs12132152), NM_000110.3:c.1236G>A (rs56038477), NC_000019.9:g.13947292T>G (rs895819) and NM_000110.3:c.1906-14763G>A (rs12022243) 12 additional variants were genotyped for the purposes of this study. Amplicons targeting these alleles were generated by multiplex PCR (primers available on request) and sequenced on the Illumina MiSeq to 100X depth using a v3 600 cycle kit (Illumina, San Diego, CA, USA). Bcl files were de-multiplexed and FASTQs generated using bcl2fastq2 (Illumina). Adapter sequences were removed using cutadapt [1], and trimmed FASTQ files were aligned to hg19 using BWA MEM, utilising the paired end read settings. Variant calling was performed using Platypus (v0.1.5), utilising the genotyping of known variants flags; –source, --minPosterior = 0, --getVariantsFromBAMs = 0, --minFlank = 0, --filterDuplicates = 0, --regions. Data QC was performed using GATK Diagnose Targets to determine variant coverage.

Call rates for all SNPs were ≥97% and all variants conformed to Hardy–Weinberg equilibrium (HWE) with the exception of rs1801266. In this case one heterozygous call and one alternate allele homozygous call were confirmed by Sanger sequencing. 

**Figure 1:**
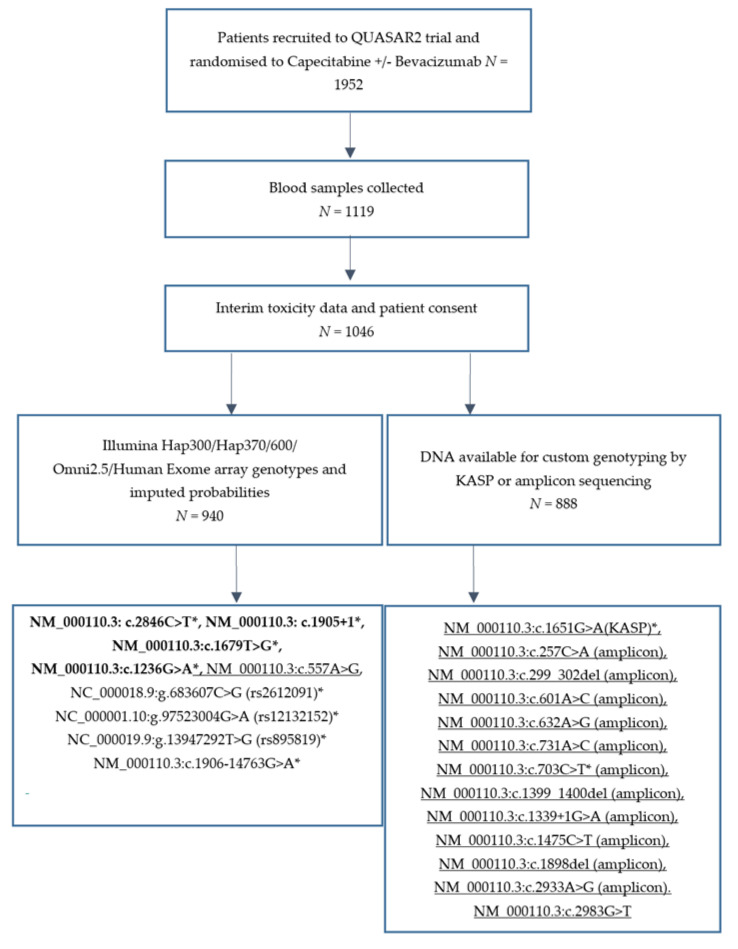
CONSORT diagram showing which QUASAR 2 samples were available for genotyping the variants meeting inclusion criteria 1 and 2. 22 Variants considered for inclusion in the panel are displayed. Those in bold have high quality evidence of a genotype to DPD phenotype in the CPIC 2018 guidelines. Variants that are underlined are the extra *DPYD* deficiency alleles under consideration based on criteria 2. The remainder are common variants fitting inclusion criteria 1. Those for which at least one variant allele was detected in QUASAR2 are marked with a *.

Adverse events related to treatment were scored at the end of each chemotherapy cycle using the NCI (National Cancer Institute) Common Terminology Criteria for Adverse Events (CTCAE) grading system and have been previously reported [23]. Toxicity data was last updated on 31 January 2016. Capecitabine-related global toxicity was coded as a binary variable (grades 0, 1, 2 versus 3 and 4). Diarrhoea, vomiting, HFS, neutropenia, thrombocytopenia and stomatitis/mucositis contributed to this variable. The ability of the tests to predict individual toxicities, coded as binary variables, was also explored. In the case of diarrhoea and haematological toxicities (thrombocytopenia and neutropenia combined) two binary codings were made so prediction of grade 4 events could be evaluated as well as prediction of grade 3/4 events. Figure 1 is a CONSORT diagram showing which samples have been included in this analysis. 

QUASAR 2 contributed to the calculation of the effect sizes of 7/10 variants detected in this study. In order to not over fit the models, data is presented where the number of risk alleles have been summed and, unless otherwise specified in the results, 1 risk allele was selected as the cut-point at which sensitivity and specificity were reported. This was done was because of the small number of patients with multiple no function/low function alleles in this dataset. The diagnostic sensitivity, diagnostic specificity, positive predictive value, negative predictive value, likelihood ratio (LR) for a positive test (LR+), and likelihood ratio for a negative test (LR−) were calculated as specified in [27]. Comparative receiver operating characteristic (ROC) curves for each model were compared using comproc in STATA (StataCorp. 2009. Stata Statistical Software: Release 11. College Station, TX, USA: StataCorp LP) specifying a normal model for calculating the percentile values. 1000 iterations of bootstrap resampling were used to generate the confidence internals for the AUCs and the Wald statistic was used to calculate the *p*-value. easyROC: A web-tool for ROC curve analysis (ver. 1.3) was used to perform power calculations. For rare events such as neutropenia we had >80% power if the area under the curve was 0.8–0.99. For the more common outcomes such as global toxicity or HFS, the study was powered to detect more modest AUCs of >0.57. Only samples with genotypes for all variants being tested were included in model/panel testing.

VariantValidator [28] was used to check the HGVS nomenclature of the variants included was valid. Where a variant can be mapped to a transcript the HGVS ID is used as the variant name, otherwise rs IDs are used.

## 3. Results

### 3.1. Markers Selected for Inclusion in a Predictive Panel

We assessed the impact of including additional variants in combination with four variants (NM_000110.3: c.2846C>T, NM_000110.3: c.1905+1, NM_000110.3:c.1679T>G, NM_000110.3:c.1236G>A) with high level evidence linking genotype to DPD activity and 5-FU toxicity in the CPIC 2018 guidelines [3]. The extra variants assessed were four common variants (rs12132152 and rs12022243 in *DPYD* [9], rs2612091 in *ENOSF1* [9] and *MIR27a* variant rs895819 [17]) which met inclusion criteria 1 and 14 *DPYD* deficiency/no function alleles which met inclusion criteria 2 (Table S1). See methods for details of genotyping. 

### 3.2. DPYD Deficiency Variants Frequency Data in QUASAR 2

Two of the 14 *DPYD* deficiency alleles genotyped were variant in QUASAR 2; NM_000110.3:c.1651G>A was detected in one individual [9] and NM_000110.3:c.703C>T was detected in two individuals. 12/14 of the *DPYD* deficiency variants genotyped in QUASAR 2 were not observed and are likely to have a frequency of <0.1% in Caucasian samples but some are more common in non-Caucasian populations (see Table S1). 

### 3.3. Comparative Receiver Operating Characteristic (ROC) Curveanalysis—Does Inclusion of Additional Variants Have Improved Diagnostic Accuracy over CPIC 2018 Variants Alone?

**Figure 2:**
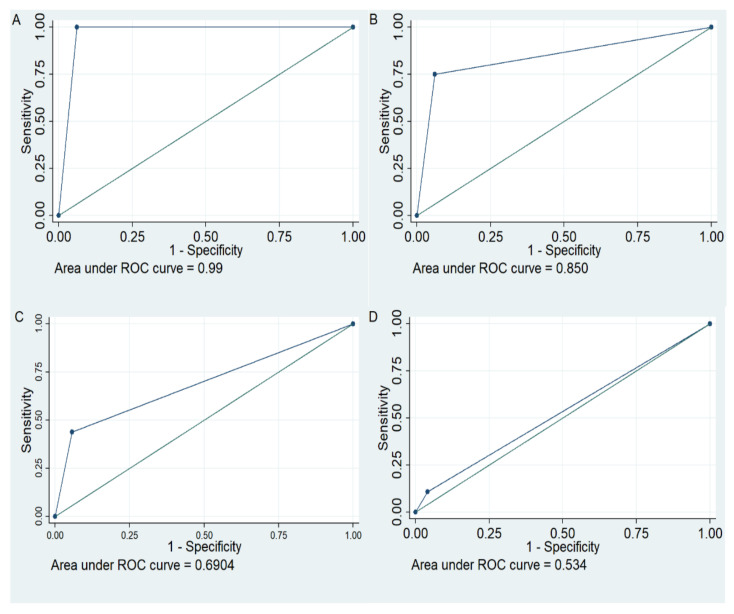
ROC curves showing performance of model 2 variants for predicting 5-Fluorouracil (5-FU) toxicity induced death, 5-FU induced haematological toxicities and global toxicity to 5-FU treatment. (**A**): Toxicity induced death, (**B**): Haematological toxicity grade 4 events, (**C**): Haematological toxicity grade 3 or 4 events, (**D**): Global toxicity.

Ten variants (Figure 1, variants marked with a *) for which at least one variant allele was present in QUASAR 2 were included in ROC analysis to examine their ability to predict risk of global and individual 5-FU related toxicities. The baseline panel of variants tested in the comparative ROC included the four variants with a high level of evidence linking genotype to DPD phenotype and 5-FU toxicity in the CPIC 2018 guidelines (NM_000110.3: c.2846C>T, NM_000110.3: c.1905+1, NM_000110.3:c.1679T>G, NM_000110.3:c.1236G>A) (Model 1, Table S2). The impact of additionally including the *DPYD* deficiency variants detected in QUASAR 2 (NM_000110.3:c.257C>A and NM_000110.3:c.703C>T) was tested (Model 2). Comparative ROC analysis using comproc showed that the AUCs were not statistically significantly different following addition of these two variants (Table 1 model 1 versus model 2). Specificity for prediction of all toxicities was retained and sensitivity for prediction of haematological toxicity (both grade 0, 1, 2 versus 3, 4 and grade 0–3 versus 4) and grade 3 mucositis/stomatitis was improved. Good diagnostic tests have an LR+ >10 and an LR− < 1. The model based on the four variants with high evidence of a link to DPD phenotype and toxicity would be defined as a good model for the prediction of toxicity induced death (based on thresholds set by Simundic [27]. The model including additional *DPYD* no function/deficiency alleles (model 2) would be defined as a good test for both toxicity-induced death and grade 4 haematological toxicity, but we note the non-significant difference in AUC between the two models when applying a bootstrapping approach to calculating confidence intervals. ROC curves showing the area under the curve with model 2 for toxicity induced death, grade 4 haematological toxicity, grade 3 haematological toxicity and global toxicity are shown in Figure 2A, B, C and D respectively. 

Having established that inclusion of extra *DPYD* deficiency alleles (model 2) did not compromise the performance of the four variants included in model 1 we tested the impact of adding each of the common SNPs (NM_000110.3:c.1906-14763G>A (model 3), rs2612091 (model 4), rs12022243 (model 5), and rs895819 (model 6)) and comparing these models to model 2. Inclusion of any of the common SNPs had a significantly detrimental impact on prediction of death and grade 4 haematological toxicity (Table 2 and Table S2) but inclusion of each of these alleles significantly improved prediction of global toxicity and or HFS and diarrhoea (Table 2). A tiered approach to the analysis of markers such that only those individuals negative for a *DPYD* deficiency allele are assessed for genotype of a set of common SNPs associated with toxicity may improve prediction of the risk of the full spectrum of 5-FU related toxicities. 

We next assessed the utility of each of the common variants included in models 3–6 for prediction of risk of diarrhoea and HFS where models 3–6 had statistically significantly better AUCs compared to model 2. (Table 3 and Table 4). The AUCs of tests containing these variants without the *DPD* deficiency associated variants and setting the cut point at 1 toxicity associated allele were compared to the AUC of model 2 (four high level evidence variants plus *DPYD* deficiency alleles detected in QUASAR 2). NM_000110.3:c.1906-14763G>A and rs895819 each improved the AUC for prediction of grade 4 and grade 3/4 diarrhoea but only rs895819 (model 7) showed a statistically significant increase in AUC for both codings of this phenotype. Sensitivity for prediction of grade 4 diarrhoea using rs895819 was 0.89 but specificity was only 0.46 and the LR+ ratio was 1.65 (Table S2). Given the low LR+ ratio we excluded this SNP for prediction of risk of diarrhoea at this time but note it may be a useful marker when combined with other markers of risk of diarrhoea discovered in the future.

Sensitivity of prediction of HFS using model 1 (four high level evidence variants) is 0.09 and specificity is 0.95. All of the common variants associated with 5-FU toxicity improved AUC for predicting grade 3 HFS (Table 2). The performance of each common variant alone (models 7–10) were compared to model 2 (Table 4 and Table S2) and two variant and three variant combinations of variants in models 8–10 (models 11–15) were also compared to model 2. AUC, sensitivity and specificity for predicting HFS risk were maximised when rs2612091 and rs12132152 were considered together with the cut-point set at 1 risk allele (sensitivity 0.84, specificity 0.31 and LR+ ratio 1.21). As can be seen in Table S2 rs2612091 and rs12132152 have to be analysed separately to the *DPYD* deficiency alleles to avoid poor prediction of the other 5-FU related toxicities.

### 3.4. Risk Categorisation of QUASAR 2 Using the Selected Variants

The variants associated with DPD deficiency performed best for prediction of risk of death and haematological grade 3 and grade 4 toxicities. Whilst 12 *DYPD* alleles meeting inclusion criteria 2 and genotyped in QUASAR 2 were not detected we expect them to be identified in larger screening populations. We therefore included them in the predictive panel design (All included variants have a “panel” ID in Table S1). rs2612091 and rs12132152 were selected to highlight risk of HFS in patients predicted to be at standard risk using the variants associated with DPD deficiency. Dosing recommendations based on the activity scores of *DPYD* genotypes have been published [3,19]. Where activity scores were not available from the CPIC *DPYD* allele functionality table (https://cpicpgx.org/guidelines/guideline-for-fluoropyrimidines-and-dpyd/, accessed on 20 February 2020) we scored the DPD deficiency associated variants based on the functional data available (Table S1). Table 5 shows the proposed risk classifications that would be provided to the clinician to help interpret the results of a test including the model 2 and model 11 variants.

Using this approach, 2 of the 888 QUASAR 2 patients genotyped would be classified as critical risk of developing toxicity; 52 as high risk; 236 would be classified as standard risk and 598 as standard risk, with high risk of HFS. The performance of this test was compared to other tests available to predict 5-FU associated toxicity (Tables S3–S5). Some of the available tests contain the four high level evidence variants highlighted by CPIC and the relative performance (AUC difference) of our final panel based on models 2 and 11 compared to this panel of variants can be seen in Table 1 and Table 4. The AUC differences when we compared our final panel to the other available tests were not significant apart from for HFS where our panel had a significantly higher AUC (*p* < 0.0001). An alternative panel consisting of the variants highlighted by CPIC plus NM_000110.3:c.1601G>A performed better (4% improvement in AUC) for the prediction of grade 4 diarrhoea (*p* < 0.0001) (Tables S4 and S5). 

### 3.5. Toxicity Outcomes of QUASAR 2 Patients Predicted to Be at Critical/High Risk of Toxicity 

Details of the toxicities and dose delays and reductions experienced by the 54 patients classified as critical or high risk of toxicity are shown in Table S6. The only two toxicity-associated deaths in QUASAR 2 occurred in these individuals. Fifty-nine percent (32/54) of the predicted critical/high risk patients experienced a grade 3/4 event (neutropenia, thrombocytopenia, diarrhoea, vomiting, mucositis/stomatitis, and hand foot syndrome) and 80% (43/54) withdrew from treatment (*n* = 15) or experienced a dose reduction and or dose delay (*n* = 28).

## 4. Discussion

Some patients experience severe, sometimes life-threatening toxicity following treatment with fluoropyrimidines. Guidelines exist for personalising dosing strategies based on *DPYD* genotype [3,19]. Current guidelines suggest testing assessing *DPYD* genotype before administering any chemotherapy including 5-FU. The DPWG and CPIC provide guidance on the dosing of 5-FU, capecitabine and tegafur without specific mention of commonly administered combination therapies including 5-FU or capecitabine such as CAPOX, FOLFOX, and FOLFIRI. A recent study demonstrated that prospective *DPYD* genotyping was feasible and improved patient safety [29]. We have generated frequency information for rare *DPYD* variants associated with DPD deficiency in a clinical trial setting in the UK population. We have shown that including additional *DPYD* deficiency variants with evidence of a deleterious impact on protein function equivalent to the CPIC high level evidence variants does not compromise the performance of a predictive panel and leads to incremental improvements in sensitivity (whilst retaining excellent specificity) for the prediction of grade 3/4 haematological toxicity, albeit with a non-significant effect on the AUC values. Addition of further *DPYD* variants identified in DPD deficient patients, with supporting evidence from approaches like *DPYD*-varifier [30], or in vitro assays measuring the impact on enzyme activity levels is likely to provide further improvements. Larger panels from diverse ethnic backgrounds will be required to determine the diagnostic accuracy of including *DPYD* deficiency alleles not observed in QUASAR 2. However, we have the proof of concept that so long as there is evidence that the variant would severely impair protein function to the level of the CPIC variants with strong evidence, then including them in a model to predict the most severe toxicities is likely to be beneficial. 

NM_000110.3:c.557A>G is very rare in European and East Asian populations and we did not detect this variant in QUASAR 2. It’s frequency in African populations is much higher (2%). Given the low cost of adding additional variants to a panel and given the functional support for the impact of this variant on DPD activity we suggest it is included to improve the performance of toxicity prediction in a broader range of ethnic backgrounds. As shown in Table S1 only 4/17 *DPYD* deficiency/no function alleles are observed in Asian populations and only 6/17 are observed in African populations (data from gnomAD v2.1.1). The predictive ability of the model 2 variants will therefore be lower in these populations and more research is required to determine whether variants detected in non-Caucasian populations are associated with toxicity. Interestingly, the risk allele of one of the common alleles associated with HFS, rs2612091, is present at a higher frequency in Asian and African populations. It has been suggested that hand foot syndrome may be more common in these populations than in white European populations [31].

Some variants have promising sensitivity and specificity for the prediction of an individual toxicity but impair sensitivity and specificity when analysed alongside *DPYD* decreased function/no function alleles. Lee et al. [32] have reported that mucositis/stomatitis and diarrhoea toxicities are often not co-incident with haematological toxicities. Whilst the reasons for toxicity in one site and not another are not fully understood it is possible that some cell types are more susceptible to the toxic effects of 5-FU than others and this may relate to differences in cell turnover in different tissues. It may be that tissues such as the mucosa and skin are more susceptible to smaller changes in the metabolism of 5-FU than neutrophils are, explaining why the common SNPs with smaller effect sizes may be useful in predicting skin and gastrointestinal toxicities but not haematological toxicity.

In our study only two individuals carried two *DPYD* deficiency/no function alleles. One was homozygous for rs1801266 and so would be predicted to have no functional DPD activity (activity score 0). The other individual carried one copy of rs3918290 and one copy of a decreased function allele rs56038477 marking HapB3 (activity score 0.5). If these variants are on different chromosomes then the patient would be expected to have no functional DPD activity but substantial activity would be retained if the two decreased function/no function alleles are on the same chromosome. Sequencing *DPYD* using long range sequencing techniques such as single molecule real time sequencing technology and Nanopore DNA sequencing would allow accurate phasing to determine whether risk alleles were on the same chromosome or not [33]. In line with current CPIC guidelines on dosing it would be recommended that that patients with activity scores of 0 or 0.5 due to possession of multiple *DPYD* deficiency/no function variants are not treated with 5-FU based chemotherapy and those with an activity score of 1 or 1.5 be given a 50% dose reduction. It may be that patients with an activity score of 1 or 1.5 who are to receive combination therapies such as FOLFOX, FOLFIRI, or CAPOX, which contain lower concentrations of 5-FU/capecitabine [34] could have their dose increased after the first few cycles if the treatment is well tolerated and indeed dose escalation has been suggested in previous publications [3] for where *DPYD* testing is implemented and activity scores of 1 or 1.5 are obtained. Guidelines for the best starting doses for 5-FU/capecitabine combination therapies in patients with activity scores between 0.5 and 1.5 are still needed to guide best patient management. 

HFS is the most common toxicity experienced by patients treated with capecitabine yet the four high level evidence variants highlighted by CPIC and DPWG have a very low sensitivity for the prediction of this toxicity (0.09) A test including rs2612091 and rs12132152 greatly improves sensitivity but at the expense of specificity (sensitivity = 0.84, specificity = 0.31). Whilst the low LR+ ratio of this test suggests it would not be useful clinically this test would allow the majority (84%) of patients at risk of grade 3 HFS to be identified so advice can be given on how to manage or prevent HFS can be given. An independent study would be required to determine whether there was a beneficial impact on quality of life of patients on capecitabine containing regimens following testing pre-treatment with 5-FU based therapy for these variants and giving advice on managing HFS such as using moisturisers or topical steroid creams.

The mechanism by which capecitabine causes HFS is not fully understood but it has been suggested that because there are higher levels of expression of Thymidine phosphorylase (TP) in the palms of the hands and the soles of the feet that a higher level of capecitabine metabolites accumulate here and lead to the symptoms of HFS [35]. Formulations of 5-FU that also include a DPD inhibitor such as S-1 (tegafur, 5-chloro-2,4-dihydroxypyridine (CDHP), potassium oxonate), uracil/tegafur (UFT) and eniluracil/5-FU treatment also have lower rates of HFS compared to capecitabine and infusional 5-FU [36]. Inhibition of DPD may lower bioavailability of metabolites causing HFS and treatments including DPD inhibitors may be useful in those known to be at high risk of HFS. The DPWG suggests that Tegafur with DPD inhibitors should only be used in those with an activity score of 2 or that where it is not possible to avoid this treatment a low dose should first be administered, and then future doses titrated guided by toxicity [19].

There are several limitations to our study. Firstly, lack of ethnic diversity; all patients included in the analysis were white Caucasian. Incidence of DPD deficiency differs between countries; whilst incidence is predicted to be 3–5% in Caucasians and 7–8% in African Americans [37] the incidence in Asian populations is much lower. One study detected DPD deficiency in only 2 in 21,200 Japanese individuals [38]. Second, the relatively small size of the cohort and the small number of serious adverse events (two deaths, four grade 4 neutropenic events, ten events of grade 4 diarrhoea). The third limitation is the use of the same dataset to examine diagnostic accuracy as was used to identify that some of the variants included in the panel were associated with toxicity. Graded toxicity data are not easily obtainable as many hospital sites do not record toxicity in a standardised way outside the clinical trial setting. Ideally diagnostic accuracy would be tested in an independent series of samples to that used to identify the markers. In the absence of such samples, we feel that an evaluation of different panels of variants in a large clinical trial such as QUASAR 2 is informative. Finally, we did not consider whether when multiple deficiency alleles were detected in an individual they were on different chromosomes.

## 5. Conclusions

We have shown that inclusion of rare *DPYD* variants identified in patients with DPD deficiency and with supporting evidence of a functional impact on DPD activity does not compromise prediction of 5-FU related toxicity and provides small improvements in the ability to predict risk of haematological toxicities. It would be interesting to try to validate this finding in a large independent sample set. We also highlight that the common variants associated with global or overall 5-FU toxicity in at least 500 patients compromise the prediction of haematological toxicities but improve the prediction of diarrhoea and HFS but not with sufficient accuracy to suggest dose modifications are made based on these tests. Management of HFS may be improved by testing for rs2612091 and rs12132152 but this would need to be confirmed in an independent study.

## 6. Patents

DK, IT, CP and DR have a patent 20170073765 pending.

## Figures and Tables

**Table 1:** AUC differences when a model containing the four variants with high evidence linking *DPYD* genotype to dihydropyrimidine dehydrogenase (DPD) phenotype and toxicity (model 1) was compared to a model containing these four variants and two additional *DPYD* deficiency alleles detected in QUASAR 2 (model 2). 845 samples were included in the analysis.

Outcome Tested	Model 1 AUC 95% CI	Model 2 AUC 95%CI	Mode 1 Versus Model 2 AUC Difference	*p*
**Models Being Compared: CPIC 2018 (Model 1), CPIC 2018 + NM_000110.3:c.257C>A +NM_000110.3:c.703C>T (Model 2).**				
Death	0.999 (0.999–1.000)	0.999 (0.999–1.000)	−0.00001 (−0.00008–0.00004)	0.52
Haematological 0123v4	0.701 (0.403–0.999)	0.850 (0.598–1.102)	0.149 (−0.108–0.407)	0.26
Haematological 012v34	0.628 (0.488–0.7690)	0.664 (0.521–0.807)	0.036 (−0.03–0.108)	0.33
Diarrhoea 0123v4	0.460 (0.348–0.573)	0.458 (0.344–0.571)	0.003 (−0.006–0.0004)	0.09
Diarrhoea 012v34	0.478 (0.438–0.517)	0.481 (0.440–0.522)	0.004 (−0.008–0.015)	0.53
Mucositis/ Stomatitis 012v3	0.587 (0.437–0.736)	0.632 (0.477–0.786)	0.045 (−0.042–0.130)	0.31
Global 012v34	0.492 (0.45–0.500)	0.482 (0.456–0.507)	0.002 (−0.004–0.009)	0.47
HFS 012v34	0.457 (0.429–0.484)	0.457 (0.429–0.485)	0.0006 (−0.007–0.008)	0.86

**Table 2:** Comparison of model 2 (DPYD deficiency alleles detected in QUASAR 2) to models additionally including a common variant previously shown to be associated with 5-FU toxicity.

Outcome	AUC Model 2	AUC Difference Model 2 vs. 3	AUC Difference Model 2 vs. 4	AUC Difference Model 2 vs. 5	AUC Difference Model 2 vs. 6
Death	0.999 (0.999−1.000)	−**0.121** **(−0.137–−0.10)** ***p* < 0.0001**	−**0.003** **(−0.005–−0.0003)** ***p* = 0.03**	−**0.269** **(−0.285–−0.253) *p* < 0.0001**	−**0.195** **(−0.212–−0.178)** ***p* < 0.0001**
Haematological 0123v4	0.850 (0.598−1.102)	0.0295 (−0.22–0.28) *p* = 0.89	−0.012 (−0.028–0.004) *p* = 0.16	−**0.289** **(−0.327–−0.251)** ***p* < 0.0001**	−**0.215** **(−0.252–−0.178)** ***p* < 0.0001**
Haematological 012v34	0.664 (0.521–0.807)	0.0463 (−0.097–0.190) *p* = 0.53	−**0.024** **(−0.035–−0.012)** ***p* < 0.0001**	−0.017 (−0.165–0.131) *p* = 0.82	−**0.113** **(−0.242–0.015))** ***p* < 0.0001**
Diarrhoea 0123v4	0.458 (0.344–0.571)	**0.217** **(0.02–0.419)** ***p* = 0.035**	−0.035 (−0.047–-0.024) ***p* < 0.0001**	−0.067 (−0.273–0.1379) *p* = 0.52	**0.269** **(0.101–0.437 )** ***p* = 0.0017**
Diarrhoea 012v34	0.481 (0.440–0.522)	**0.109** **(0.0390–0.179)** ***p* = 0.0023**	−0.025 (−0.045–−0.005) *p* = 0.014	0.098 (0.036–0.160) *p* = 0.002	0.154 (0.086–0.222) ***p* < 0.0001**
Mucositis/Stomatitis012v3	0.632 (0.477–0.786)	0.144 (−0.0246–0.314) *p* = 0.094	0.024 (−0.064–0.112) *p* = 0.59	−0.004 (−0.178–0.169) *p* = 0.96	0.072 (−0.105–0.249) *p* = 0.42
Global 012v34	0.482 (0.456–0.507)	**0.123** **(0.077−0.169)** ***p* < 0.0001**	**0.030** **(0.008−0.053)** ***p* = 0.008**	**0.141** **0.100–0.182)** ***p* < 0.0001**	**0.075** **(0.027–0.122)** ***p* = 0.0021**
HFS 012v34	0.457 (0.429–0.485)	**0.129** **(0.079–0.180)** ***p* < 0.0001**	**0.044** **(0.015−0.073)** ***p* = 0.0027**	**0.159** **(0.112–0.207)** ***p* < 0.0001**	**0.057** **(0.003–0.112)** ***p* = 0.039**

Models being compared: CPIC 2018 + NM_000110.3:c.257C>A +NM_000110.3:c.703C>T (model 2) vs. CPIC 2018 + NM_000110.3:c.257C>A +NM_000110.3:c.703C>T + NM_000110.3:c.1906-14763G>A (model 3), CPIC 2018 + NM_000110.3:c.257C>A +NM_000110.3:c.703C>T + rs12132152 (model 4), CPIC 2018 + NM_000110.3:c.257C>A +NM_000110.3:c.703C>T + rs2612091 (model 5) CPIC 2018 + NM_000110.3:c.257C>A +NM_000110.3:c.703C>T + rs895819 (model 6). 845 samples were included in the comparison of models 2–5 and 790 samples were included in the comparison of models 2 and 6. rs895819 was imputed in QUASAR 2 and hard coded genotypes were generated for those with a genotype probability >0.9 (*n* = 790), samples will lower probabilities were marked as missing for this variant. Comparisons yielding a statistically significant increase in AUC are highlighted in bold.

**Table 3:** Comparative ROC analysis of models for the prediction of diarrhoea.

Outcome Tested	Model 2 AUC 95% CI	AUC Difference Model 2 Versus Model 7	AUC Difference Model 2 Versus 8
Diarrhoea 0123v4	0.458 (0.344–0.571)	**0.28** **(0.103–0.459)** ***p* = 0.002**	0.173 (−0.094–0.440) *p* = 0.2
Diarrhoea 012v34	0.481 (0.440–0.522)	**0.131** **(0.051–0.210)** ***p* = 0.0012**	**0.084** **(0.003–0.166)** ***p* = 0.04**

rs895819 (model 7) and rs12022243 (model 8) for prediction of diarrhoea. *n* samples = 790 for model 7 and 845 for model 8. Results that reach significance at *p* < 0.05 are highlighted in bold.

**Table 4:** Comparative ROC analysis of models for the prediction of HFS.

Model Number	AUC Model 2	AUC Difference Compared to Model 2
7	0.461 (0.301–0.492)	−0.036 (−0.00267–0.099) *p* = 0.26
8	0.457 (0.429–0.486)	0.090 (0.025–0.155) *p* = 0.007
9	0.457 (0.429–0.486)	0.157 (0.107–0.208) *p* < 0.0001
10	0.457 (0.429–0.486)	0.036 (−0.005–0.077) *p* = 0.085
11	0.457 (0.429–0.486)	0.177 (0.129–0.226) *p* < 0.0001
12	0.457 (0.429–0.486)	0.164 (0.117–0.210) *p* < 0.0001
13	0.457 (0.429–0.486)	0.122 (0.057–0.186) *p* = 0.0002
14	0.457 (0.429–0.486)	0.174 (0.128–0.219) *p* < 0.0001
15	0.457 (0.429–0.486)	0.161 (0.100–0.222) *p* < 0.0001

Model 7 = rs895819, model 8 = NM_000110.3:c.1906-14763G>A (rs12022243), model 9 = rs2612091, model 10 = rs12132152, model 11 = rs2612091 + rs12132152, model 12 = rs2612091 + rs12022243, model 13 = rs12022243 + rs12132152, model 14 = rs2612091 + rs12022243 + rs12132152 cutpoint 1, model 15 = rs2612091 + rs12022243 + rs12132152 cutpoint 2. 790 samples were included in the analysis of model 7 and 845 samples in the analysis of models 8–15.

**Table 5:** Proposed risk classifications for a panel made up of model 2 and model 11 variants.

Status	Genotype	Results	Clinical Interpretation
Critical RISK	A patient carries two no-function alleles or one no function and one low function allele	The DPD activity score prediction is 0 or 0.5. The test indicates for this individual that they are of Critical Risk as they have variants that indicate DPD Deficiency.	For patients identified as CRITICAL RISK and therefore possibly DPD DEFICIENT you should avoid use of 5-FU or 5-FU prodrug-based regimens.
High RISK	A patient carries one copy of a no-function allele or one or two copies of a decreased function allele	The DPD activity score is 1 or 1.5. This individual is predicted to have at least 2× the risk of grade 3/4 toxicity using a standard dose of capecitabine or 5-FU monotherapy in comparison to the Standard Risk group. The variants detected are strongly associated with Partial DPD Deficiency.	For patients identified as HIGH RISK, a 5-FU or 5-FU prodrug-based regimen dose modulation of 50% is recommended. Consider dose titration guided by toxicity after first 2 cycles.
Standard RISK	A patient carries no copies of any no function/deceased function alleles or any HFS-associated allele	The DPD activity score is 2. The test indicates no increased risk of grade 3/4 toxicity using a standard dose of capecitabine or 5-FU monotherapy in comparison to the Standard Risk.	For patients identified as STANDARD RISK, with no other contradicting factors there is no indication to change dose or therapy. Use label recommended dosage and administration.
Standard RISK High Risk HFS	A patient carries no copies of a no function/decreased function allele, but one or more allele(s) associated with increased risk of HFS	The DPD activity score is 2. The test indicates no increased risk of grade 3/4 toxicity using a standard dose of capecitabine or 5-FU monotherapy in comparison to the Standard Risk. However, there is a high risk of HFS, this risk is at least 2× the risk of the Standard Risk Population.	For patients identified as STANDARD RISK with HIGH RISK HFS there is no indication to change dose or therapy. Use label recommended dosage and administration Advice on how to minimise/prevent HFS according to local guidelines is recommended.

## Data Availability

The data presented in this study are available on request from the corresponding author.

## Supplementary Materials

The following are available online at https://www.mdpi.com/2072-6694/13/7/1497/s1, Table S1: Genetic markers considered for inclusion in a predictive panel, Table S2: Measures of diagnostic accuracy used to select a panel of variants for predicting 5-FU induced toxicity, Table S3: Genetic markers included in other tests to predict toxicity if treated with capecitabine and other fluoropyrimidine based therapy, Table S4: Measures of diagnostic accuracy of model 2 and model 11 compared to alternative testing panels currently available in the UK, Table S5: Comparison of the performance of model2 and model 11 to available commercial tests that are not identical to the CPIC/DPWG high level evidence set of four variants, Table S6: Details of patients classified as high risk or critical risk by model 2.
