# Supplementary material for: An Evaluation of the Diagnostic Accuracy of a Panel of Variants in DPYD and a Single Variant in ENOSF1 for Predicting Common Capecitabine Related Toxicities
Source: Cancers (Basel). 2021 Mar 24;13(7):1497. doi: 10.3390/cancers13071497 (PMC8037940; doi:10.3390/cancers13071497)
Supplement: Supplementary file 1 [file cancers-13-01497-s001.pdf]

Supplementary Materials

An Evaluation of the Diagnostic Accuracy of a Panel of Variants in *DPYD* and a Single Variant in ENOSF1 for Predicting Common Capecitabine Related Toxicities

Claire Palles, Susan Fotheringham, Laura Chegwidden, Marie Lucas, Rachel Kerr, Guy Mozolowski, Dan Rosmarin, Jenny C. Taylor, Ian Tomlinson and David Kerr

Table S1. Genetic markers considered for inclusion in a predictive panel. Those in models 2 and 11 have a panel variant number.

Final Panel Variant Number	Variant ID HGVS Nomenclature Protein Change	Associated with Global Fluoroprimidine Toxicity	Detected in a DPD Deficiency Patient (Reference)	Evidence from Literature	CPIC Level of Evidence	Per Allele Activity Score 1 Activity Score 2 Activity Score 3	In DPYD LOVD with Pathogenic/Risk/Likely Pathogenic Scoring	Allele Frequency in QUASAR 2	Allele Frequency in 1KG	Allele Frequency in gnomAD^ Europeans (non-Finnish)	Allele Frequency in gnomAD East Asia	Allele Frequency in gnomAD Africa	Method of Genotyping
1	rs67376798 NM_000110.3: c.2846C>T p.D949V	Yes OR 5.51 (95% CI 1.95–15.5, p = 0.0013)	Yes[1]	Risk allele results in 59% DPYD activity compared to wild type and or 5-FU toxicity [2,3]. **CPIC guideline variant**	High	0.5 PHENO NA	yes	0.006	NA	5.16×10^{-3}	5.30×10^{-5}	9.16×10^{-4}	Genotyped on the Illumina Exome array
2	rs3918290 NM_000110.3: c.1905+1 G>A	Yes OR 5.51 (95% CI 1.95–15.5, p = 0.0013)*	Yes[4–6]	Catalytically inactive allele [2,7] **CPIC guideline variant**	High	/0 0 NA	yes	0.004	NA	8.97×10^{-6}	0	0	Genotyped on the Illumina Exome array
3	rs12132152 NC_000001.10: g.97523004G>A	Yes OR 3.83 (95% CI 3.26–4.40) p = 3.29 × 10	No	Common variant associated with HFS [8]	Not reviewed	Not scored – common variant	no	0.028	0.06	0.028	0	8.48×10^{-3}	genotyped (Illumina array 620 and omni 2.5), imputed from hap 370
5	rs777425216 NM_000110.3:c. 1651G>A p.A551T		Yes[4]	This mutation might prevent binding of the prosthetic group FMN and affect folding of the DPYD protein [4]	Weak	not scored not scored 0	no	0.006	NA	0	5.80×10^{-5}	0	Custom KASP assay
6	rs2612091 NC_000018.9:g. 683607C>G	Yes OR 1.59, 95% CI 1.45–1.94, p = 2.55 × 10^{-5}	No	Common variant associated with Hand-foot syndrome [8]	Not reviewed	Not scored – common variant	no	0.552	NA	0.554	0.71	0.79	Genotyped on all Illumina arrays

7	rs568132506 NM_000110.3:c. 257C>A p.P86L	Yes [1]	Analysis of the crystal structure of pig DPYD suggests that the missense variant 257C>T; Pro86Leu interferes directly or indirectly with cofactor binding or electron transport [1]	Weak	Not scored Not scored 0	no	monomorphic	NA	5.53 × 10⁻⁵	0	0	New genotyping for this study, done by amplicon sequencing
8	rs72549309 NM_000110.3:c. 299_302del p.F100fs	Yes [5]	The deletion allele was associated with undetectable DPYD activity [3]	Weak	0Not scored NA	yes	monomorphic	NA	0	0	0	New genotyping for this study, done by amplicon sequencing
9	rs72549308 NM_000110.3:c. 601A>C p.S201R	Yes [1]	Crystal structure of pig DPYD suggests this missense variant interferes directly or indirectly with cofactor binding or electron transport [1]	Weak	0Not scored NA	no	monomorphic	NA	5.38 × 10⁻⁵	0	6.54 × 10⁻⁵	New genotyping for this study, done by amplicon sequencing
10	rs72549307 NM_000110.3:c. 632A>G p.Y211C	Yes [1]	Identified in homozygous state in a patient with DPYD deficiency. Analysis of the crystal structure of pig DPYD suggests that the missense variant destabilises DPYD protein [1].	Weak	0Not scored NA	no	monomorphic	NA	8.97 × 10⁻⁶	0	0	New genotyping for this study, done by amplicon sequencing
11	rs1185250556 NM_000110.3:c. 731A>C p.E244A	Yes [4]	Mutation might interfere with the electron flow between NADPH and the pyrimidine binding site of DPYD [4]	Not reviewed	Not scored Not scored 0	no	monomorphic	NA	1.79 × 10⁻⁵	0	0	New genotyping for this study, done by amplicon sequencing

12	rs1801266 NM_000110.3:c.703C>T p.R235W		Yes[5]	Identified in compound heterozygous state in a patient with DPYD deficiency[6]. T allele associated with reduced DPYD activity [3]	Weak	0Not scored NA	no	0.002	NA	9.48×10^{-5}	5.30×10^{-5}	0	New genotyping for this study, done by amplicon sequencing
13	1039-1042delTG NM_000110.3:c.1399_1400delp.P466fs		Yes[1]	Frameshift mutation expected to effect NADPH binding leading to an inactive protein[9]	Not reviewed	Not scored Not scored 0	no	monomorphic	NA	0	0	0	New genotyping for this study, done by amplicon sequencing
14	IVS11+1G>T NM_000110.3:c.1339+1G>A		Yes[4]	Mutation created a cryptic splice site within exon 11. Amino acids 400–446 of DPYD were missing from the mature DPYD mRNA. No DPYD activity [4] detected in homo-zygous individual.	Not reviewed	Not scored Not scored 0	no	monomorphic	NA	0	0	0	New genotyping for this study, done by amplicon sequencing
15	rs72549304 NM_000110.3:c.1475C>T p.S492L		Yes[1]	Analysis of the crystal structure of pig DPYD suggests the missense variant interferes directly or indirectly with cofactor binding or electron transport [1]	Weak	0Not scored NA	no	monomorphic	NA	0	0	3.33×10^{-4}	New genotyping for this study, done by amplicon sequencing
16	rs55886062 NM_000110.3:c.1679T>G p.I560S	Yes RR 4·40, 95% CI 2·08–9·30, $p < 0.0001$	Yes[1]	Allele results in low DPYD activity and or 5-FU toxicity [2,7] **CPIC guideline variant**	High	00NA	yes	0.0006	NA	6.11×10^{-4}	0	8.33×10^{-5}	Genotyped using Illumina exome array

17	rs72549303 NM_000110.3:c. 1898del p.P633fs		Yes[6]	Allele results in undetectable DPYD activity[3] and or 5-FU toxicity [2]	Weak	0 Not scored NA	no	monomorphic	NA	0	0	0	New genotyping for this study, done by amplicon sequencing
19	rs72547601 NM_000110.3:c. 2933A>G p.H978R		Yes[1]	Analysis of the crystal structure of pig DPYD suggests the missense variant interferes directly or indirectly with cofactor binding or electron transport [1]	Weak	0 Not scored NA	no	monomorphic	NA	0	0	0	New genotyping for this study, done by amplicon sequencing
20	rs1801268 NM_000110.3:c. 2983G>T p.V995F		Yes[9]	This missense change is located in a region thought to be involved in the binding of a [4Fe-4 S] cluster[9]	Weak	0	no	monomorphic	NA	0	0	0	New genotyping for this study, done by amplicon sequencing
21	rs115232898 NM_000110.3:c. 557A>G p.Y186C		Yes[10]	Allele results in low DPYD activity and or 5-FU toxicity [7]. It has only been observed in heterozygous state and only in African Americans[11]	Moderate	0.5 Not scored NA	no	monomorphic	NA	6.33×10^{-5}	5.30×10^{-5}	0.022	Genotyped using Illumina exome array
22	rs56038477 NM_000110.3:c. 1236G>A p.E412E	Yes HR 1·59, 95% CI 1·29–1·97, p < 0·0001	No*	In LD with deep intronic SNP which creates a cryptic spice site[12] **CPIC guideline variant**	Strong	0.5 PHENO NA	yes	0.020	0.022	0.021	2.51×10^{-4}	0.003	Imputed
	rs895819 NC_000019.9:g. 13947292T>G	Yes OR 1.6, 95% CI: 1.10–2.22, p = 0.012	No	SNP increases expression of MIR27a which reduces DPYD expression[13]	Not reviewed	Not scored – common variant	no	0.320	0.328	0.327	0.276	0.5014	Imputed. Not analysed before
	rs12022243 NM_000110.3:c. 1906-14763G>A	Yes OR 1.69, 95% CI 1.45–1.94, $p = 2.55 \times 10^{-5}$	No	Common SNP associated with global fluoropyrimidine toxicity[8]	Not reviewed	Not scored – common variant	no	0.205	0.187	0.190	0.248	0.1640	Genotyped (Illumina array 620 and omni 2.5), imputed from hap 370

^ Frequency data obtained from gnomAD v2.1.1. Note Panel variant numbers go from 1–22 but there is no variant 4 or 18. * results presented were from a combined analysis of rs3918290 and rs67376798. Activity score 1: Activity scores for per variant allele [14] and listed in https://api.pharmgkb.org/v1/download/file/attachment/DPYD_Allele_Functionality_Table.xlsx (last accessed 04/10/2020). Activity score 2: Activity scores per allele using grading system according to DPWG [15]. For

this score the authors include the category “PHENO” which means phenotyping by measuring DPD enzyme activity in peripheral blood mononuclear cells (PBMCs) or to measuring the uracil concentrations in plasma or urine is recommended by DPWG Activity score 3: Where no activity score had been calculated for a variant we scored variants based on their association with DPD deficient phenotype and the available supporting functional evidence. Functional DPD alleles would score 1 in all three scores. For heterozygous individuals with one copy of a DPD deficiency variant allele their activity score would be 1+ the scores shown in Table S1 and this score can be used to determine the correct dosing recommendation from CPIC and DPWG publications [15,16].

Table S2. Measures of diagnostic accuracy used to select a panel of variants for predicting 5-FU induced toxicity.

Model Number	Test	Toxicity Outcome	True Negative	False Negative	False Positive	True Positive	Sensitivity	Specificity	AUC	LR+	LR-
1	CPIC 2018 High level of evidence variants	Toxicity induced Death	794	0	49	2	1.00	0.94	0.97	17.20	0.00
1	CPIC 2018 High level of evidence variants	Haematological 012v34	784	10	45	6	0.38	0.95	0.66	6.91	0.66
1	CPIC 2018 High level of evidence variants	Haematological 0123v4	792	2	49	2	0.50	0.94	0.72	8.58	0.53
1	CPIC 2018 High level of evidence variants	Diarrhoea 012v34	705	89	39	12	0.12	0.95	0.53	2.27	0.93
1	CPIC 2018 High level of evidence variants	Diarrhoea 0123v4	786	9	50	1	0.10	0.94	0.52	1.67	0.96
1	CPIC 2018 High level of evidence variants	Mucositis/Stomatitis 012v3	785	9	47	4	0.31	0.94	0.63	5.45	0.73
1	CPIC 2018 High level of evidence variants	Global 012v34	539	255	22	29	0.10	0.96	0.53	2.60	0.93
1	CPIC 2018 High level of evidence variants	HFS 012v34	622	172	35	16	0.09	0.95	0.52	1.60	0.97
2	CPIC 2018 plus rs777425216 and rs1801288	Toxicity induced Death	791	0	52	2	1.00	0.94	0.97	17.04	0.00
2	CPIC 2018 plus rs777425216 and rs1801288	Haematological 012v34	782	9	47	7	0.44	0.95	0.69	8.11	0.59
2	CPIC 2018 plus rs777425216 and rs1801288	Haematological 0123v4	790	1	51	3	0.75	0.94	0.84	13.00	0.27
2	CPIC 2018 plus rs777425216 and rs1801288	Diarrhoea 012v34	703	88	41	13	0.13	0.95	0.54	2.46	0.92
2	CPIC 2018 plus rs777425216 and rs1801288	Diarrhoea 0123v4	782	9	53	1	0.10	0.94	0.52	1.65	0.96
2	CPIC 2018 plus rs777425216 and rs1801288	Mucositis/Stomatitis 012v3	783	8	49	5	0.38	0.94	0.66	6.36	0.66
2	CPIC 2018 plus rs777425216 and rs1801288	Global 012v34	536	252	23	31	0.11	0.96	0.53	2.84	0.93
2	CPIC 2018 plus rs777425216 and rs1801288	HFS 012v34	617	170	37	17	0.09	0.94	0.52	1.61	0.96
**Model 2 was selected in favour of the CPIC 2018 variants (model 1)**											
3	Model 2 plus rs12022243	Toxicity induced Death	487	0	356	2	1.00	0.58	0.79	2.37	0.00
3	Model 2 plus rs12022243	Haematological 012v34	483	4	346	12	0.75	0.58	0.67	1.80	0.43
3	Model 2 plus rs12022243	Haematological 0123v4	487	0	354	4	1.00	0.58	0.79	2.38	0.00
3	Model 2 plus rs12022243	Diarrhoea 012v34	443	44	301	57	0.56	0.60	0.58	1.39	0.73
3	Model 2 plus rs12022243	Diarrhoea 0123v4	484	3	351	7	0.70	0.58	0.64	1.67	0.52
3	Model 2 plus rs12022243	Mucositis/Stomatitis 012v3	485	2	347	11	0.85	0.58	0.71	2.03	0.26
3	Model 2 plus rs12022243	Global 012v34	360	127	201	157	0.55	0.64	0.60	1.54	0.70
3	Model 2 plus rs12022243	HFS 012v34	402	85	255	103	0.55	0.61	0.58	1.41	0.74
4	Model 2 plus rs12132152	Toxicity induced Death	747	0	96	2	1.00	0.89	0.94	8.78	0.00
4	Model 2 plus rs12132152	Haematological 012v34	738	9	91	7	0.44	0.89	0.66	3.99	0.63
4	Model 2 plus rs12132152	Haematological 0123v4	746	1	95	3	0.75	0.89	0.82	6.64	0.28
4	Model 2 plus rs12132152	Diarrhoea 012v34	661	86	83	15	0.15	0.89	0.52	1.33	0.96
4	Model 2 plus rs12132152	Diarrhoea 0123v4	738	9	97	1	0.10	0.88	0.49	0.86	1.02
4	Model 2 plus rs12132152	Mucositis/Stomatitis 012v3	740	7	92	6	0.46	0.89	0.68	4.17	0.61
4	Model 2 plus rs12132152	Global 012v34	520	227	41	57	0.20	0.93	0.56	2.75	0.86
4	Model 2 plus rs12132152	HFS 012v34	597	150	60	38	0.20	0.91	0.56	2.21	0.88

5	Model 2 plus rs2612091	Toxicity induced Death	232	0	611	2	1.00	0.28	0.64	1.38	0.00
5	Model 2 plus rs2612091	Haematological 012v34	230	2	599	14	0.88	0.28	0.58	1.21	0.45
5	Model 2 plus rs2612091	Haematological 0123v4	231	1	610	3	0.75	0.27	0.51	1.03	0.91
5	Model 2 plus rs2612091	Diarrhoea 012v34	209	23	535	78	0.77	0.28	0.53	1.07	0.81
5	Model 2 plus rs2612091	Diarrhoea 0123v4	227	5	608	5	0.50	0.27	0.39	0.69	1.84
5	Model 2 plus rs2612091	Mucositis/Stomatitis 012v3	230	2	602	11	0.85	0.28	0.56	1.17	0.56
5	Model 2 plus rs2612091	Global 012v34	178	54	383	230	0.81	0.32	0.56	1.19	0.60
5	Model 2 plus rs2612091	HFS 012v34	197	35	460	153	0.81	0.30	0.56	1.16	0.62
6	Model 2 plus rs895819	Toxicity induced Death	335	0	463	2	1.00	0.42	0.71	1.72	0.00
6	Model 2 plus rs895819	Haematological 012v34	329	6	455	10	0.63	0.42	0.52	1.08	0.89
6	Model 2 plus rs895819	Haematological 0123v4	334	1	462	3	0.75	0.42	0.58	1.29	0.60
6	Model 2 plus rs895819	Diarrhoea 012v34	311	24	396	69	0.74	0.44	0.59	1.32	0.59
6	Model 2 plus rs895819	Diarrhoea 0123v4	334	1	457	8	0.89	0.42	0.66	1.54	0.26
6	Model 2 plus rs895819	Mucositis/Stomatitis 012v3	333	2	454	11	0.85	0.42	0.63	1.47	0.36
6	Model 2 plus rs895819	Global 012v34	235	100	299	166	0.62	0.44	0.53	1.11	0.85
6	Model 2 plus rs895819	HFS 012v34	262	73	364	101	0.58	0.42	0.50	1.00	1.00
**Models 3–6 were rejected based upon the reduced specificity of predicting toxicity induced death and haematological toxicities. Next examined clinical utility of common variants associated with toxicity to predict diarrhoea and HFS risk separately**											
7	rs895819	Diarrhoea 012v34	340	29	373	63	0.68	0.48	0.58	1.31	0.66
7	rs895819	Diarrhoea 0123v4	368	1	428	8	0.89	0.46	0.68	1.65	0.24
8	rs12022243	Diarrhoea 012v34	505	51	243	45	0.47	0.68	0.57	1.44	0.79
8	rs12022243	Diarrhoea 0123v4	552	4	282	6	0.60	0.66	0.62	1.77	0.60
8	rs12022243	HFS 012v34	450	106	237	88	0.45	0.66	0.56	1.31	0.83
9	rs2612091	HFS 012v34	218	40	468	153	0.79	0.32	0.55	1.16	0.65
10	rs12132152	HFS 012v34	662	168	25	25	0.13	0.96	0.56	3.56	0.90
11	rs2612091+rs12132152	HFS 012v34	212	31	474	161	0.84	0.31	0.57	1.21	0.52
12	rs2612091+rs12022243	HFS 012v34	144	23	542	170	0.88	0.21	0.54	1.11	0.57
13	rs12022243+rs12132152	HFS 012v34	440	93	247	100	0.52	0.64	0.58	1.44	0.75
14	all three	HFS 012v34	148	20	543	175	0.90	0.21	0.56	1.14	0.48
15	all three (cut point 2)	HFS 012v34	425	81	266	114	0.58	0.62	0.60	1.52	0.68
**Model including rs2612091 and rs12132152 has too low specificity to be clinically useful for predicting non HFS toxicities**											
16	Model 2 and Model 11 combined	Toxicity induced Death	220	0	622	2	1.00	0.26	0.6306	1.35	0.00
16	Model 2 and Model 11 combined	Haematological 012v34	218	2	610	14	0.88	0.26	0.5691	1.19	0.47
16	Model 2 and Model 11 combined	Haematological 0123v4	219	1	621	3	0.75	0.26	0.5054	1.01	0.96
16	Model 2 and Model 11 combined	Diarrhoea 012v34	198	22	545	79	0.78	0.27	0.5243	1.07	0.82
16	Model 2 and Model 11 combined	Diarrhoea 0123v4	215	5	619	5	0.50	0.26	0.3789	0.67	1.94
16	Model 2 and Model 11 combined	Mucositis/Stomatitis 012v3	218	2	613	11	0.85	0.26	0.5542	1.15	0.59
16	Model 2 and Model 11 combined	Global 012v34	175	45	386	238	0.84	0.31	0.5765	1.22	0.51
16	Model 2 and Model 11 combined	HFS 012v34	192	28	465	159	0.85	0.29	0.5713	1.20	0.51

Models being compared: NM_000110.3: c.2846C>T, NM_000110.3: c.1905+1, NM_000110.3:c.1679T>G, NM_000110.3:c.1236G>A) (CPIC 2018, model 1), CPIC 2018 + NM_000110.3:c.257C>A +NM_000110.3:c.703C>T (model 2) vs CPIC 2018 + NM_000110.3:c.257C>A +NM_000110.3:c.703C>T + NM_000110.3:c.1906-14763G>A (model 3), CPIC 2018 + NM_000110.3:c.257C>A +NM_000110.3:c.703C>T + rs12132152 (model 4) , CPIC 2018 + NM_000110.3:c.257C>A +NM_000110.3:c.703C>T + rs2612091 (model 5) CPIC 2018 + NM_000110.3:c.257C>A +NM_000110.3:c.703C>T + rs895819 (model 6), rs895819 (model 7), NM_000110.3:c.1906-14763G>A (rs12022243) (model 8), rs2612091 (model 9), rs12132152 (model 10), rs2612091+ rs12132152 (model 11), rs2612091+ rs12022243 (model 12), rs12022243+ rs12132152 (model 13), rs2612091+ rs12022243+ rs12132152 cut point 1 (model 14), rs2612091+ rs12022243+ rs12132152 cut point 2 (model 15).

Table S3. Genetic markers included in other tests to predict toxicity if treated with capecitabine and other fluoropyrimidine based therapy.

Panel Designation	Genetic Markers Included (no.)	Genetic Marker Details	Genetic Markers Unique to This Test when Compared to Model 2	Dosing Recommendations Summary	Notable Test Feature
Model 2	20 (18 general toxicity)	see Table S1 for details	N/A	Yes, see Table 2 for details	2 genetic markers included associated with risk of HFS
CPIC 2013 guideline markers	3	rs67376798, rs3918290, rs55886062,	N/A	Yes[2]	
CPIC 2018 guideline markers (same as model 1)	4	rs67376798, rs3918290, rs55886062, rs75017182 (c.1129-5923C>G/HapB 3)	rs75017182 (a proxy rs56038477 is included in Model 2 which in perfect LD in with rs75017182)	This provider [1] with a test based on these markers returns predicted DPYD enzyme activity for all 4 variants (0% for rs3918290 and 55886062 and 50% for rs67376798 and HapB3) and makes reference to CPIC 2018 clinical guidelines	One provider of this test updated website to include recommendation that genotype of the decreased function variant rs115232898 (c.557A>G) p.(Tyr186Cys) in individuals with African ancestry should be assessed
CPIC 2018 and rs1801158	5	rs67376798, rs3918290, rs55886062, rs56038477 rs1801158	rs1801158	Provider [2] recommends dose reductions or alternative therapy for rs3918290 and rs55886062 (50%), rs67376798 and HapB3 (25%). Dose reduction of 20% and followed by incremental dose increase for rs1801158. No fluoropyrimidines for homozygote rs3918290 and 75% dose for any other compound heterozygotes	
CPIC 2013 and rs72549309	4 (+phenotype testing see notes)	rs67376798, rs3918290, rs72549309, rs55886062	N/A	Yes, recommendations based on combination of genotype and phenotype test [3].	Phenotypic testing combining testing of pre-treatment plasma levels of the endogenous natural uracil substrate (U) and its metabolite dihydrouracil (UH2).

[1] https://www.exeterlaboratory.com/genetics/prediction-of-5-fluorouracil-toxicity/ (last accessed 04/10/2020); [2] http://www.viapath.co.uk/our-tests/mutation-analysis-dpd-for-5-fluorouracil-toxicity (last accessed 04/10/2020); [3] https://www.eurofins.ie/biomnis/latest-news/dpd-deficiency-testing-and-therapeutic-drug-monitoring/ (last accessed 04/10/2020).

Table S4. Measures of diagnostic accuracy of model 2 and model 11 compared to alternative testing panels currently available in the UK.

Test	Toxicity Outcome	True Negative	False Negative	False Positive	True Positive	Sensitivity	Specificity	NPV	PPV	AUC	LR+	LR-
Model 2	Toxicity induced Death	791	0	52	2	1.00	0.94	1.00	0.04	0.97	16.21	0.00
	Neutropenia 012 v 34	782	9	47	7	0.44	0.94	0.99	0.13	0.69	7.72	0.60
	Neutropenia 0123 v 4	790	1	51	3	0.75	0.94	1.00	0.06	0.84	12.37	0.27
	Diarrhoea 012 v 34	703	88	41	13	0.13	0.94	0.89	0.24	0.54	2.34	0.92
	Diarrhoea 0123 v 4	782	9	53	1	0.10	0.94	0.99	0.02	0.52	1.58	0.96
	Mucositis/ Stomatitis_012v3	783	8	49	5	0.38	0.94	0.99	0.09	0.66	6.53	0.65
	Global 012 v 34	536	252	23	31	0.38	0.94	0.99	0.09	0.53	6.53	0.65
	HFS 012 v 34	617	170	37	17	0.11	0.96	0.68	0.57	0.52	2.66	0.93
Model 11	HFS 012 v 34	212	31	474	161	0.84	0.31	0.87	0.25	0.57	1.21	0.52
CPIC 2013 and NM_000110.3:c.299_302del (rs72549309)	Toxicity induced Death	848	0	16	2	1.00	0.98	1.00	0.11	0.99	54.00	0.00
	Neutropenia 012 v 34	837	11	13	5	0.31	0.98	0.99	0.28	0.65	20.43	0.70
	Neutropenia 0123 v 4	846	2	16	2	0.50	0.98	1.00	0.11	0.74	26.94	0.51
	Diarrhoea 012 v 34	749	98	14	4	0.04	0.98	0.88	0.22	0.51	2.14	0.98
	Diarrhoea 0123 v 4	838	9	17	1	0.10	0.98	0.99	0.06	0.54	5.03	0.92
	Mucositis/ Stomatitis_012v3	836	12	16	2	0.14	0.98	0.99	0.11	0.56	7.61	0.87
	Global 012 v 34	575	273	6	12	0.04	0.99	0.68	0.67	0.52	4.08	0.97
	HFS 012 v 34	663	184	13	5	0.03	0.98	0.78	0.28	0.50	1.38	0.99
CPIC 2018 and NM_000110.3:c.1601G>A (rs1801158)	Toxicity induced Death	754	0	84	2	1.00	0.90	1.00	0.02	0.95	9.98	0.00
	Neutropenia 012 v 34	744	10	80	6	0.38	0.90	0.99	0.07	0.64	3.86	0.69
	Neutropenia 0123 v 4	752	2	84	2	0.50	0.90	1.00	0.02	0.70	4.98	0.56
	Diarrhoea 012 v 34	671	83	70	16	0.16	0.91	0.89	0.19	0.53	1.71	0.93
	Diarrhoea 0123 v 4	747	7	84	2	0.22	0.90	0.99	0.02	0.56	2.20	0.87
	Mucositis/ Stomatitis_012v3	745	9	82	4	0.31	0.90	0.99	0.05	0.60	3.10	0.77
	Global 012 v 34	514	240	44	42	0.15	0.92	0.68	0.49	0.54	1.89	0.92
	HFS 012 v 34	591	163	62	24	0.13	0.91	0.78	0.28	0.52	1.35	0.96

Some of the tests available in the UK only contain the four variants with high level evidence in the CPIC 2018 review and recommended for clinical testing by DPWG. The performance of these four variants versus the panel finally selected (panel 2 and panel 11 SNPs) can be seen in Table 2. NPV = negative predictive value; PPV = positive predictive value; AUC = area under the curve.

Table S5. Comparison of the performance of model2 and model 11 to available commercial tests that are not identical to the CPIC/DPWG high level evidence set of four variants.

Outcome Tested	Model 2 (or 11 for HFS) AUC 95% CI	AUC Difference Model 2/11 versus CPIC 2013 and NM_000110.3:c.299_302del (rs72549309) (95%CI) *p*	AUC Difference Model 2/11 versus CPIC 2018 and NM_000110.3:c.1601G>A (rs1801158) (95%CI) *p*
Death	0.999 (0.999–1.000)	−0.001 (−0.003–0.0002) *p* = 0.09	0.00004 (−0.0001–0.0002) *p* = 0.61
Haematological 0123v4	0.850 (0.592–1.107)	−0.166 (−0.430–0.098) *p* = 0.22	−0.128 (−0.388–0.132) *p* = 0.34
Haematological 012v34	0.664 (0.522–0.806)	−0.057(−0.129–0.014) *p* = 0.12	−0.043 (−0.1389–0.053) *p* = 0.38
Diarrhoea 0123v4	0.464 (0.338–0.591)	0.045 (-0.085–0.174) *p* = 0.5	**0.040 (0.025–0.054) *p* < 0.0001**
Diarrhoea 012v34	0.483 (0.440–0.525)	0.475 (0.427–0.522) *p* = 0.59	−0.154 (−0.050–0.020) *p* = 0.39
Mucositis/Stomatitis 012v3	0.631 (0.472–0.790)	−0.068 (−0.161–0.26) *p* = 0.16	−0.102 (−0.238–0.033) *p* = 0.14
Global 012v34	0.482 (0.456–0.508)	−0.005 (−0.0257–0.0149) *p* = 0.6	0.002 (−0.021–0.024) *p* = 0.88
HFS 012v34	0.634 (0.594–0.674)	**−0.184 (−0.232–−0.135) *p* < 0.0001**	**−0.175(−0.221–−0.130) *p* < 0.0001**

Model 2 variants are used to predict all toxicities apart from HFS. Model 11 is used to predict HFS only.

Table S6. Details of patients classified as high risk or critical risk by model 2.

					CTCAE Grade Event								
ID	Risk Genotype(s)	Activity Score ψ	Cycles Received	Toxicity Induced Death	Thrombocytopenia	Neutropenia	Diarrhoea	HFS	Vomiting	Mucositis/Stomatitits	Other Side Effects (grade/cycle(s))	Cycle when dose Reduction made, % Reduction	Dose Delay (Cycle, Delay in Weeks)
					CRITICAL RISK PREDICTION								
13	rs1801266 homozygote	0	8		0	0	1	3	0	1		2,25,4,25,6,25	2,1,3,1
14	rs3918290 heterozygote, HapB3 heterozygote	0.5	1	1	4	4	0	3	0	3	Respiratory failure secondary to neutropenia.	NA	Withdrew after/ during first cycle
					HIGH RISK PREDICTION								
1	rs1801266 heterozygote	1	8		0	0	1	1	0	1	Fatigue (3/1), dizziness (not graded/ 2)	3,25	3,1
2	rs3918290 heterozygote	1	1		0	0	2	0	0	0	Dehydration (3/1)	NA	Withdrew after/ during first cycle
3	rs55886062 heterozygote	1	8		0	0	1	2	0	1	Skin rash on face (not graded/1), Fatigue (not graded/3)	2,25, 4,25	no delays
4	rs67376798 heterozygote	1	8		0	3	1	1	0	0		2,25	2,3, 3,1
5	rs3918290 heterozygote	1	3		0	0	0	3	0	0		2,20	2,1,3,1
6	rs67376798 heterozygote	1	6		0	0	1	3	0	1		2,50 *	7,2

7	rs3918290 heterozygote	1	8		0	0	2	1	1	0		No reductions	2,4
8	rs67376798, heterozygote	1	8		1	3	2	1	0	0		3,25	2,3,3,3,7,2
9	rs3918290 heterozygote	1	8		0	0	1	2	0	0	Recurrent HFS (2/3,4)	5,30	no delays
10	rs67376798 heterozygote	1	2		0	0	3	0	0	0		No reductions	withdrew after 2 cycles
11	rs67376798, heterozygote	1	8		0	0	2	1	0	1		No reductions	no delays
12	rs3918290 heterozygote	1	8		0	0	1	3	0	0		4,22	4,2
15	rs67376798 heterozygote	1	1		0	0	3	0	2	0		NA	withdrew after/during first cycle
16	rs67376798 heterozygote	1	8		0	0	4	0	0	1		2,50	2,1
17	rs67376798 heterozygote	1	1	1	4	4	2	1	−9	2	Neutropenic colitis and left ventricular hypertrophy.	NA	Withdrew after/during first cycle
18	rs777425216 heterozygote	1	1		4	4	3	2	0	3		NA	Withdrew after/during first cycle
19	rs67376798 heterozygote	1	8		0	2	3	1	0	2		4,25	2,1,4,2
20	rs3918290 heterozygote	1	1		0	3	3	0	0	0		NA	Withdrew after/during first cycle
21	rs67376798 heterozygote	1	1		0	0	1	3	3	3		NA	Withdrew after/during first cycle
22	HapB3 heterozygote	1.5	8		0	0	0	2	0	0		0	no delays
23	HapB3 heterozygote	1.5	8		0	0	1	3	0	1	Fatigue (1,3)	4,25	no delays
24	HapB3 heterozygote	1.5	8		0	0	1	0	0	1	Skin rash (1,1), muscle/joint pain (1,2), fatigue (1,3), dry skin (1,3)	0	no delays
25	HapB3 heterozygote	1.5	8		0	0	2	3	0	0	Tremor (2,2)	3,30	no delays
26	HapB3 heterozygote	1.5	8		0	0	1	3	0	1	Fatigue (1,1), glucoseuria/hyper-glycemia (1,1)	2,25,3,25	
27	HapB3 heterozygote	1.5	8		0	0	1	3	0	0	Fatigue (1,2), dry, sore eyes (1,1), liver dysfunction (1,5), peripheral neuropathy (1,7)	4,25	
28	HapB3 heterozygote	1.5	8		0	0	1	3	0	0			no delays

29	HapB3 hetero-zygote	1.5	2	0	1	2	1	2	0	Fatigue (2,2)	stopped after 2 cycles -bowel obstruction	no delays
30	HapB3 hetero-zygote	1.5	8	0	2	2	3	1	0		6,25	4, 1
31	HapB3 hetero-zygote	1.5	8	0	0	1	1	0	1	Anxiety (1,1), cystitis (2,4), fatigue (1,8)	No reductions	no delays
32	HapB3 hetero-zygote	1.5	4	0	0	0	0	0	1	Died of other causes during cycle 4	No reductions	no delays
33	HapB3 hetero-zygote	1.5	8	0	0	0	1	0	1	Fatigue (2,1), DVT (3,5), liver dysfunction (1,5)	1 week delay due to DVT	no delays
34	HapB3 hetero-zygote	1.5	8	0	0	0	1	0	1	Fatigue (1,3), peripheral neuropathy (1,3)	No reductions	no delays
35	HapB3 hetero-zygote	1.5	8	1	2	0	3	0	1	Altered taste (1,1), dry skin (1,1), sore eyes (1,2), anorexia (1,5)	7,25	no delays
36	HapB3 hetero-zygote	1.5	7	1	1	3	1	3	1	Infection, pulmonary embolism (4,7)	2,25	2,1
37	HapB3 hetero-zygote	1.5	8	0	0	1	1	1	0	Fatigue (1,5)	No reductions	no delays
38	HapB3 hetero-zygote	1.5	3	0	0	0	1	0	0	Shortness of breath/cough (1,1) loss of appetite (1,2), general pain (1,2)	Patient decision to withdraw due to side effects and caring commitments	
39	HapB3 hetero-zygote	1.5	8	1	0	0	1	0	0	Dry skin/hair (1,1), fatigue (1,7) constipation (1,7)	No reductions	no delays
40	HapB3 hetero-zygote	1.5	8	0	0	2	3	0	3	Sore eyes (1,2)	2,25	2,1
41	HapB3 hetero-zygote	1.5	8	0	0	2	2	0	0	Anaemia (1,1), DVT foot (2,5),	No reductions	8,2
42	HapB3 hetero-zygote	1.5	8	0	0	2	0	0	1		3,33	
43	HapB3 hetero-zygote	1.5	8	0	0	1	1	0	1	Fatigue(1,6)	No reductions	no delays
44	HapB3 hetero-zygote	1.5	2	0	3	3	0	3	1		2,25. Patient withdrew after cycle 2 due to toxicity	Patient stopped after 2 cycles due to toxicity
45	HapB3 hetero-zygote	1.5	8	0	2	3	2	2	2		2,30	2,1 and 5,1
46	HapB3 hetero-zygote	1.5	3	0	0	3	1	1	1	Liver dysfunction (1,5), sore eyes (1,7)	4,30	4,1
47	HapB3 hetero-zygote	1.5	2	0	0	3	1	1	1	Upper body rash (1,2)	patient withdrew due to toxicity	2,1
48	HapB3 hetero-zygote	1.5	7	0	0	3	3	0	1	Miscellaneous pain (2,2)	3,25%	Last cycle omitted due to HFS
49	HapB3 hetero-zygote	1.5	8	0	0	0	0	0	0	Fatigue (1,4), reduced renal function leading to dose reduction	2,17%	no delays

50	HapB3 heterozygote	1.5	3	0	0	3	3	0	2		2,25%	2,1. Patient stopped capecitabine after 3 cycles due to toxicity
51	HapB3 heterozygote	1.5	1	0	0	2	3	0	3	Sore eyes (3,1)	2,100% - patient withdrawn due to toxicity	
52	HapB3 heterozygote	1.5	8	0	1	3	2	2	2	Fatigue (1,3), anorexia (3,1)	3,22%, 4,33%	
53	HapB3 heterozygote	1.5	1	0	0	0	0	0	0	Myocardial infarction, chest pain as symptom (4,1)	1,100%-patient withdrawn because of cardiac symptoms	
54	HapB3 heterozygote	1.5	1	0	0	0	0	0	0	Chest pain (1,2)	1, 100%	

References

1. Van Kuilenburg, A.B.; Dobritzsch, D.; Meinsma, R.; Haasjes, J.; Waterham, H.R.; Nowaczyk, M.J.; Maropoulos, G.D.; Hein, G.; Kalhoff, H.; Kirk, J.M.; et al. Novel disease-causing mutations in the dihydropyrimidine dehydrogenase gene interpreted by analysis of the three-dimensional protein structure. *Biochem. J.* **2002**, *364*, 157–163, doi:10.1042/bj3640157.
2. Caudle, E.K.; Thorn, C.F.; Klein, T.E.; Swen, J.J.; McLeod, H.L.; Diasio, R.B.; Schwab, M. Clinical pharmacogenetics implementation consortium guidelines for dihydropyrimidine dehydrogenase genotype and fluoropyrimidine dosing. *Clin. Pharmacol. Ther.* **2013**, *94*, 640–645.
3. Offer, S.M.; Fossum, C.C.; Wegner, N.J.; Stuflesser, A.J.; Butterfield, G.L.; Diasio, R.B. Comparative functional analysis of DPYD variants of potential clinical relevance to dihydropyrimidine dehydrogenase activity. *Cancer Res.* **2014**, *74*, 2545–2554, doi:10.1158/0008-5472.can-13-2482.
4. Van Kuilenburg, A.B.; Meinsma, R.; Beke, E.; Bobba, B.; Boffi, P.; Enns, G.M.; Witt, D.R.; Dobritzsch, D. Identification of three novel mutations in the dihydropyrimidine dehydrogenase gene associated with altered pre-mRNA splicing or protein function. *Biol. Chem.* **2005**, *386*, 319–324.
5. Van Kuilenburg, A.B.P.; Vreken, P.; Abeling, N.G.G.M.; Bakker, H.D.; Meinsma, R.; Van Lenthe, H.; De Abreu, R.A.; Smeitink, J.A.M.; Kayserili, H.; Apak, M.Y.; et al. Genotype and phenotype in patients with dihydropyrimidine dehydrogenase deficiency. *Qual. Life Res.* **1999**, *104*, 1–9, doi:10.1007/pl00008711.
6. Vreken, P.; Van Kuilenburg, A.B.P.; Meinsma, R.; Van Gennip, A.H. Dihydropyrimidine dehydrogenase (DPD) deficiency: Identification and expression of missense mutations C29R, R886H and R235W. *Qual. Life Res.* **1997**, *101*, 333–338, doi:10.1007/s004390050637.
7. Offer, S.M.; Wegner, N.J.; Fossum, C.; Wang, K.; Diasio, R.B. Phenotypic profiling of DPYD variations relevant to 5-fluorouracil sensitivity using real-time cellular analysis and in vitro measurement of enzyme activity. *Cancer Res.* **2013**, *73*, 1958–1968, doi:10.1158/0008-5472.can-12-3858.
8. Rosmarin, D.; Palles, C.; Pagnamenta, A.; Kaur, K.; Pita, G.; Martin, M.; Domingo, E.; Jones, A.; Howarth, K.; Freeman-Mills, L.; et al. A candidate gene study of capecitabine-related toxicity in colorectal cancer identifies new toxicity variants at DPYD and a putative role for ENOSF1 rather than TYMS. *Gut* **2015**, *64*, 111–120.
9. Morel, A.; Boisdron-Celle, M.; Fey, L.; Soulie, P.; Craipeau, M.C.; Traore, S.; Gamelin, E. Clinical relevance of different di-hydropyrimidine dehydrogenase gene single nucleotide polymorphisms on 5-fluorouracil tolerance. *Mol. Cancer Ther.* **2006**, *5*, 2895–9204.
10. Ezzeldin, H.H.; Lee, A.M.; Mattison, L.K.; Diasio, R.B. Methylation of the DPYD promoter: An alternative mechanism for dihydropyrimidine dehydrogenase deficiency in cancer patients. *Clin. Cancer Res.* **2005**, *11*, 8699–8705, doi:10.1158/1078-0432.ccr-05-1520.
11. Saif, M.W.; Lee, A.M.; Offer, S.M.; McConnell, K.; Relias, V.; Diasio, R.B. A DPYD variant (Y186C) specific to individuals of African descent in a patient with life-threatening 5-FU toxic effects: Potential for an individualized medicine approach. *Mayo Clin. Proc.* **2014**, *89*, 131–136, doi:10.1016/j.mayocp.2013.09.008.
12. Meulendijks, D.; Henricks, L.M.; Van Kuilenburg, A.B.P.; Jacobs, B.A.W.; Aliev, A.; Rozeman, L.; Meijer, J.; Beijnen, J.H.; De Graaf, H.; Cats, A.; et al. Patients homozygous for DPYD c.1129-5923C>G/haplotype B3 have partial DPD deficiency and require a dose reduction when treated with fluoropyrimidines. *Cancer Chemother. Pharmacol.* **2016**, *78*, 875–880, doi:10.1007/s00280-016-3137-0.

13. Offer, S.M.; Butterfield, G.L.; Jerde, C.R.; Fossum, C.C.; Wegner, N.J.; Diasio, R.B. MicroRNAs miR-27a and miR-27b directly regulate liver dihydropyrimidine dehydrogenase expression through two conserved binding sites. *Mol. Cancer Ther.* **2014**, *13*, 742–751, doi:10.1158/1535-7163.mct-13-0878.
14. Henricks, L.M.; Lunenburg, C.A.; Meulendijks, D.; Gelderblom, H.; Cats, A.; Swen, J.J.; Schellens, J.H.; Guchelaar, H.J. Translating DPYD genotype into DPD phenotype: Using the DPYD gene activity score. *Pharmacogenomics* **2015**, *16*, 1277–1286.
15. Lunenburg, C.A.T.C.; Van Der Wouden, C.H.; Nijenhuis, M.; Rhenen, M.H.C.-V.; De Boer-Veger, N.J.; Buunk, A.M.; Houwink, E.J.F.; Mulder, H.; Rongen, G.A.; Van Schaik, R.H.N.; et al. Dutch pharmacogenetics working group (DPWG) guideline for the gene–drug interaction of DPYD and fluoropyrimidines. *Eur. J. Hum. Genet.* **2019**, *28*, 508–517, doi:10.1038/s41431-019-0540-0.
16. Amstutz, U.; Henricks, L.M.; Offer, S.M.; Barbarino, J.; Schellens, J.H.; Swen, J.J.; Klein, T.E.; McLeod, H.L.; Caudle, K.E.; Diasio, R.B.; et al. Clinical pharmacogenetics implementation consortium (CPIC) guideline for dihydropyrimidine dehydrogenase genotype and fluoropyrimidine dosing: 2017 update. *Clin. Pharmacol. Ther.* **2018**, *103*, 210–216, doi:10.1002/cpt.911.
